# Protocol for a cluster randomised controlled trial of LPG cookstoves compared to usual cooking practices to reduce perinatal mortality and morbidity in rural Bangladesh called *Poriborton*: the CHANge trial

**DOI:** 10.1186/s13063-022-06146-7

**Published:** 2022-04-18

**Authors:** Camille Raynes-Greenow, Ashraful Alam, Sk Masum Billah, Sajia Islam, Kingsley Agho, S. M. Rokonuzzaman, Jonathan Thornburg, Shams El Arifeen, Atique Iqbal Chowdhury, The Poriborton Trial Team, Bin Jalaludin, Nick Goodwin, Alison Hayes, Tanvir Huda, Md. Jahiduj Jaman, Jasmin Khan, Michael J. Dibley

**Affiliations:** 1 The University of Sydney, Sydney School of Public Health, Camperdown, Australia; 2 Maternal and Child Health Division, icddr, Dhaka, Bangladesh; 3 School of Science and Health, Western Sydney University, Sydney, Australia; 4 RTI International, Research Triangle Park, NC 27707 USA

**Keywords:** Household air pollution, Perinatal mortality, Bangladesh, Cluster randomised controlled trial

## Abstract

**Background:**

Household air pollution is a leading health risk for global morbidity and mortality and a major health risk in South Asia. However, there are no prospective investigations of the impact of household air pollution on perinatal morbidity and mortality. Our trial aims to assess the impact of liquefied petroleum gas (LPG) for cooking to reduce household air pollution exposure on perinatal morbidity and mortality compared to usual cooking practices in Bangladesh.

**Hypothesis:**

In a community-based cluster randomised controlled trial of pregnant women cooking with LPG throughout pregnancy, perinatal mortality will be reduced by 35% compared with usual cooking practices in a rural community in Bangladesh.

**Methods:**

A two-arm community-based cluster randomised controlled trial will be conducted in the Sherpur district, Bangladesh. In the intervention arm, pregnant women receive an LPG cookstove and LPG in cylinders supplied throughout pregnancy until birth. In the control or usual practice arm, pregnant women continue their usual cooking practices, predominately traditional stoves with biomass fuel. Eligible women are pregnant women with a gestational age of 40–120 days, aged between 15 and 49 years, and permanent residents of the study area. The primary outcome is the difference in perinatal mortality between the LPG arm and the usual cooking arm. Secondary outcomes include (i) preterm birth and low birth weight, (ii) personal level exposure to household air pollution, (iii) satisfaction and acceptability of the LPG stove and stove use, and (iv) cost-effectiveness and cost-utility in reducing perinatal morbidity and mortality. We follow up all women and infants to 45 days after the birth. Personal exposure to household air pollution is assessed at three-time points in a sub-sample of the study population using the MicroPEM™. The total required sample size is 4944 pregnant women.

**Discussion:**

This trial will produce evidence of the effectiveness of reduced exposure to household air pollution through LPG cooking to reduce perinatal morbidity and mortality compared to usual cooking practices. This evidence will inform policies for the adoption of clean fuel in Bangladesh and other similar settings.

**Trial registration:**

Australian New Zealand Clinical Trials Registry ACTRN12618001214224. Prospectively registered on 19 July 2019

**Supplementary Information:**

The online version contains supplementary material available at 10.1186/s13063-022-06146-7.

## Background

Globally, household air pollution is a leading health risk for mortality, contributing up to approximately 4.3 million deaths or 7.7% of all deaths per year [1, 2]. Approximately 3 billion people depend on solid fuels (e.g. wood, dung, crop waste) for cooking, and this, combined with incomplete combustion using inefficient stoves, in poorly ventilated areas, over long hours of cooking and heating, causes exposure to household air pollution [3]. The South Asian burden is acute, with approximately 74% of households depending on solid fuels for cooking [4]. The established disease profile includes acute respiratory infection, ischaemic heart disease, and lung cancer [2]. However, there are no comprehensive investigations or confirmation of the impact of household air pollution on perinatal mortality [5]. Pregnant women and children are at greater risk due to a combination of their higher vulnerability and their higher levels of exposure due to domestic responsibilities [6–8]. There is some epidemiological evidence of the effect of household air pollution on perinatal outcomes, including three published systematic reviews [5, 8, 9]. The most recent review examined the impact on stillbirth and low birth weight [9]. The reported summary-effect estimate on stillbirth was 1.29 (95% *CI* 1.18, 1.41), and for low birth weight, 1.35 (95% *CI* 1.23, 1.48) [9]. Overall, all reviews report low quality of the included studies due to the mostly retrospective designs with a high risk of information and selection bias, unclear definitions of stillbirth with small sample sizes, considerable variability in the exposure assessment, insufficient adjustment for confounders, and the most recent review noted evidence of publication bias [8–12]. The association for early neonatal mortality is also not clearly understood from these studies.

Perinatal mortality is a major global public health problem [13, 14]. Globally, neonatal mortality and stillbirth are extraordinarily high, with approximately 2.5 million global neonatal deaths a year and ~2.6 million stillbirths per year [13]. South Asia has the second-highest stillbirth burden and accounts for almost 40% of all stillbirths [13]. In Bangladesh, the stillbirth rate is ~35 per 1000 births, and worse in rural areas [15, 16], where there is almost universal reliance on polluting fuels [17–19].

In low-resource settings, studies quantifying household air pollution exposure have reported pollutant levels in houses that far exceed the WHO acceptable levels [20]. In Bangladesh, exposure to biomass fuel emissions from cooking is very high, especially in rural settings. Three studies from Bangladesh, using data from the Bangladesh Demographic and Health Surveys, have described the use of solid fuel for cooking as high as 93%, and associations between exposure from cooking with solid fuels and adverse perinatal outcomes such as stillbirth [21, 22], early neonatal mortality [22], and low birth weight [23]. Cleaner fuels such as liquefied petroleum gas (LPG) are relatively available and scalable and considered an acceptable intervention to reduce exposure to household air pollution [24]. We developed a community-based cluster randomised controlled trial to assess the impact of cooking with LPG compared to usual cooking (predominantly traditional biomass fuel stoves) on perinatal mortality in pregnant women in rural Bangladesh. The trial is called Poriborton: The CHANge trial (*Poriborton*, a Bangla word meaning ‘change for better’, and *C*lean *H*ousehold *A*ir for *N*ewborns trial, ACTRN12618001214224).

## Methods

### Aims and hypothesis

The primary aim is to assess the impact of LPG cooking in reducing perinatal mortality compared to usual cooking practices in rural Bangladesh. The secondary aims are to evaluate the effect of LPG cooking compared to usual cooking on neonatal mortality, preterm birth, and low birth weight. Our objective is to assess the reduction of exposure to household air pollution in the LPG cooking arm compared to usual cooking and determine the cost-effectiveness of LPG cooking compared to usual cooking in reducing perinatal morbidity and mortality.

We hypothesise that in a community-based, cluster randomised controlled trial of women in early pregnancy from a rural community in Bangladesh, the use of LPG will reduce perinatal mortality (stillbirth and early neonatal mortality) by 35% compared with usual cooking practices. We defined stillbirth from ≥28 weeks gestation and early neonatal mortality up to 7 days after birth [25].

### Study design

The design is a parallel community-based cluster randomised controlled superiority trial (cRCT) of two arms. The intervention arm (i) includes an LPG cookstove and LPG cylinders with behaviour change communication, provided from early pregnancy until birth, and (ii) the control arm, continues usual cooking practices. The usual cooking practice in this setting is a traditional clay stove with various biomass solid fuels [26].

### Study setting

**Fig. 1:**
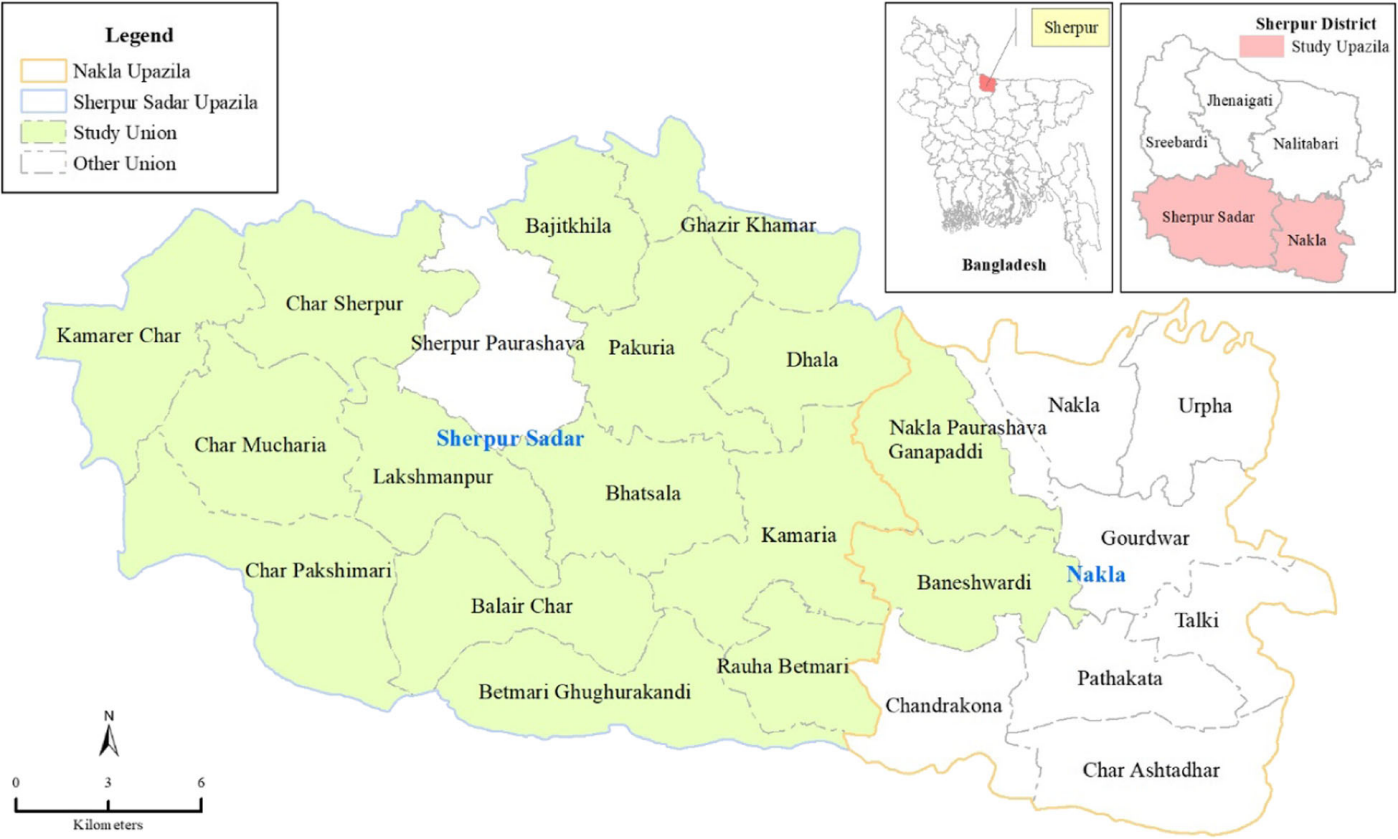
Sherpur, the Poriborton study site, in Northern Bangladesh, divided by Unions (main picture). Insert (left): Bangladesh divided by districts with Sherpur (red). Insert (right): the selected sub-districts (red)

The study will be in the Sherpur district of Mymensingh Division, Bangladesh (Fig. 1). Sherpur is approximately 200 km north of Dhaka. The district has been selected based on the high neonatal mortality rate (46 per 1000 live births) and the low penetration of LPG fuel for cooking [19]. Sherpur has five sub-districts and a population of ~1,170,219 living in rural areas. Out of the five sub-districts, we will purposively select two for the study based on the road conditions that allow a good connection to the district headquarters for LPG transportation and study staff access. A total of 16 Unions (the smallest administrative unit) will be selected from the two chosen sub-districts considering (a) the community reliance on traditional cooking practices, (b) geographically not prone to flooding during the monsoonal season, and (c) a similar intervention is not ongoing in that area.

### Design, randomisation, and intervention allocation

We define a cluster as a geographical area of ~ 445 households or ~ 1800 people. According to the 2011 population census of Bangladesh [27], the average population size per union in Sherpur is ~25,000 people yielding up to 20 clusters per Union. The field team will conduct an initial listing of residents in the study area to get the exact size of the population. We will then select 206 clusters and apply a block randomisation to allocate the clusters with a 1:1 ratio [28], resulting in 103 clusters per arm and an equal number of intervention and control clusters from each union. We will generate random blocks of sequential 1 and 2 using Stata SE [29], where each number represents either the intervention or the control arm. Non-investigators will allocate the clusters to the treatment arms based on the random sequence.

**Fig. 2:**
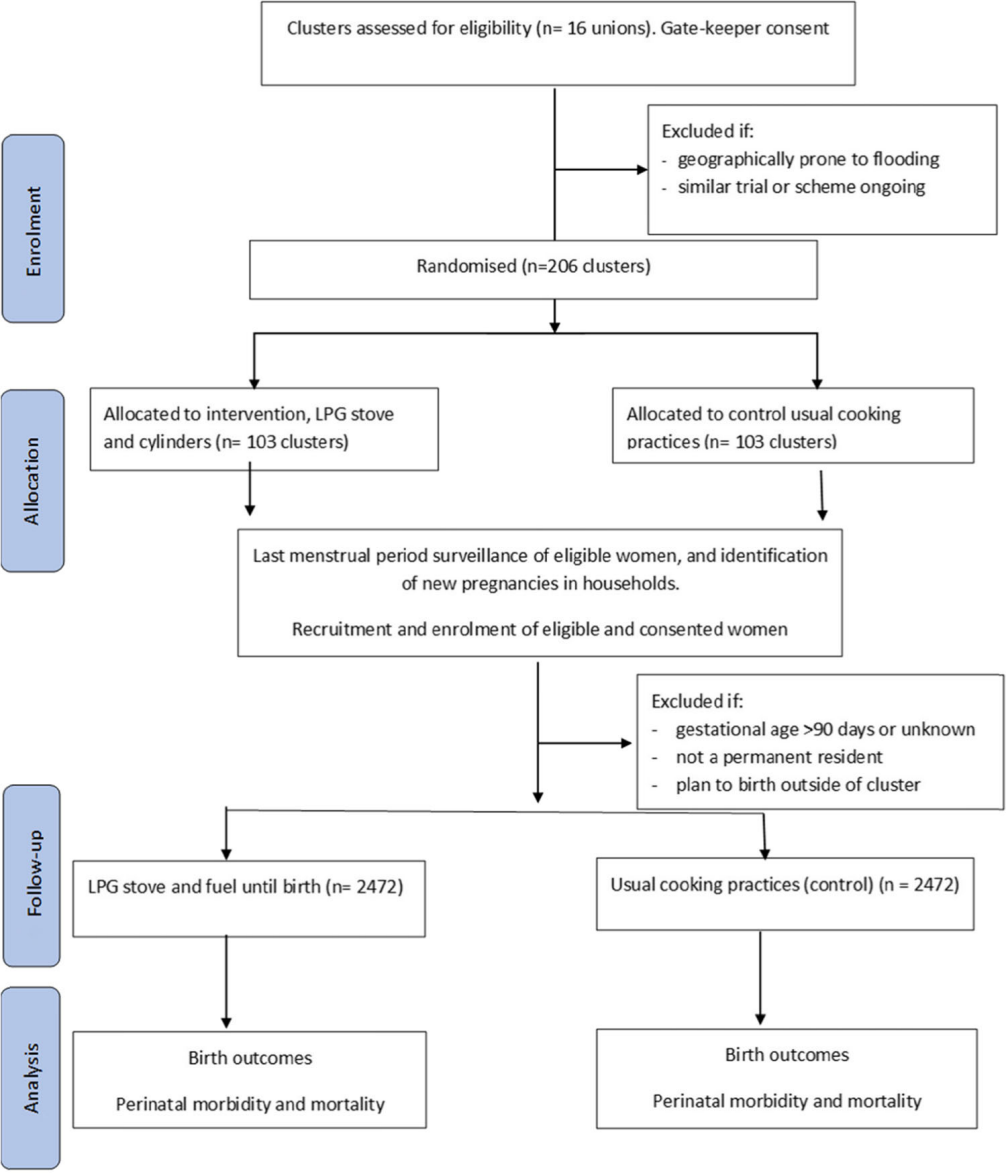
Poriborton trial flow diagram

To minimise bias, because participant blinding is not possible, we will have separate intervention and evaluation staff. Trial evaluators will be blinded to the hypothesis, and we will conduct our analysis blinded to the treatment arms. The intervention is distributed at the individual level but randomised at the cluster level to reduce community tensions and overall exposure and leverage community-level adoption of clean cooking. We will conduct outcome assessment at the individual level. Allocation concealment is impractical due to the cluster design and nature of the intervention. Pregnant women and their households receive the intervention based on their cluster of residence, and LPG cylinder supply will continue through to birth (Fig. 2).

### Study population

We have two levels of inclusion and exclusion criteria, and the first level is for cluster eligibility. Eligible clusters are those with a predominantly rural population. Exclusion criteria are (i) clusters already participating in a stove scheme or are within or sharing boundaries of areas with a relatively high existing use of LPG cooking and (ii) clusters prone to flooding for extended periods. The second level for eligibility is for women. Eligible women are (i) between 15 and 49 years of age, (ii) pregnant with a gestational age between 40 and 120 days determined by self-reported date of the last menstrual period, (iii) permanent residents of the study area, and (iv) planning to give birth in their cluster of residence. Exclusion criteria include (i) women whose gestational age cannot be determined, (ii) pregnancy loss before receipt of the intervention, and (iii) women who are not permanent residents of the study area.

### Feasibility study

Before finalising the trial design, we conducted a single-arm non-randomised feasibility study of LPG stove and cylinder distribution [30]. The findings from this study had multiple outcomes that identified issues and improved processes for conducting our trial: firstly, the development of a locally appropriate LPG cylinder supply chain in the study area, and secondly, the development of content relevant for our initial behaviour change communication such as benefits of clean cooking, practical suggestions for modifying cooking behaviours, and the important role of mothers-in-law and husbands in decision-making about cooking and fuel practices [30]. Lastly, we confirmed culturally appropriate approaches for measuring personal exposure to household air pollution and produced representative data that confirmed the LPG intervention did reduce exposure to cooking fuel emissions [31].

### Formative research

We conducted formative research to more specifically inform the behaviour change communication materials. Experienced qualitative researchers trained in ethnographic methods stayed in selected households in the trial area to observe the cooking behaviour of the family. The researcher collected extensive field notes of their observations and conducted interviews with the person mainly responsible for cooking in the family to generate data on families’ everyday cooking behaviours and related aspects, current cooking practices, and the role of persons within the family and the community in cooking-related decision-making. In addition, we performed direct observation of the cooking environment, including the kitchen and any other places used for cooking and fuel storage. We conducted focus groups with women to generate information about the women’s perceptions and experiences of usual cooking practices and adopting LPG stoves for cooking. This was done to develop culturally appropriate and attractive messages for the community to support consistent and correct use of LPG cooking during the intervention period (manuscript in preparation).

### Intervention

The LPG stove will have a double burner, and we will use a 15-kg cylinder. The Bangladesh Standard and Testing Institute have accredited both the stove and cylinder. The stove company provides logistic support, maintenance, and technical troubleshooting throughout the study period. Our field implementation team are trained in technical issues related to gas stove set-up, ignition, connection and use, troubleshooting, maintenance, and cylinder replacement.

The behaviour change communication messages are designed to promote and support individual and household adoption of correct and consistent LPG cooking. The intervention field staff will distribute the behaviour change communication material and support the participants and their household members in the intervention arm once a month to promote consistent use of LPG cooking during pregnancy. They will also discuss practical stove use issues and safety. The materials include a calendar and a leaflet with pictorial messages and three short movies, plus a fourth focused solely on safety played on a tablet device. The scripts for the short movies will be iteratively written by our team based on the formative research and professionally produced by the gas supplier using local actors performing in the dialect from the study site. These movies will follow the story of a household that would be considered typical in our study area who purchase an LPG stove and cylinder for the newly pregnant woman . Each movie is a slightly different story for each pregnancy trimester and will run for approximately 5–10 min.

To ensure continuous LPG coverage, we have collaborated with the gas supplier to establish a supply chain for the project. The participants will request an LPG replacement cylinder via our intervention worker (either by phone or in-person) who enters the request into the Web-based system specifically developed for this trial, which will be accessible in the field using electronic tablets. The intervention worker will then forward the notification of the request to the respective gas storage hub manager (there are approximately 1–2 of these per Union). The participant or her representative will collect the replacement cylinder from the hub manager within 24 h after the initial request, which we will track using our real-time tablet system.

### Recruitment

#### Identification and enrolment of eligible women and supply of LPG stove and cylinder

We will have three types of field workers for study implementation: surveillance staff, intervention staff, and evaluation (outcome) staff. The surveillance staff will identify eligible women and conduct door-to-door visits to the households in both the intervention and control areas in two monthly cycles. In the initial round, the surveillance staff will prepare a comprehensive list of all existing currently married women in the study clusters (pregnancy outside marriage is not common in the study area). They will then prospectively visit the listed married women every alternate month until the required number of pregnancies are identified and enrolled from each cluster. At each contact, currently married women of reproductive age will report their last menstrual cycle date. Any woman whose date of her last menstrual cycle is >40 days will be recorded as potentially pregnant. Pregnancy will be confirmed by a urine test strip kit (Exel®). Once a woman is identified and recorded as pregnant on our Web-based data collection platform, the system will send an automatic notification to the evaluation worker. The evaluation worker will visit the pregnant woman and, following written informed consent, will enrol the woman. Our door-to-door surveillance of ~92,000 households, over an anticipated period of 12 months, is expected to identify and enrol the required number of pregnant women within 120 days of gestational age.

A list of all enrolled women from the intervention clusters will be sent to the intervention facilitators. Within 72 h of the enrolment notification, the intervention facilitators will visit the women to provide detailed information on the gas stove, introduce the participant or her representative to the local gas storage hub manager, and explain the process of collecting the stove and cylinder. The intervention facilitators will also ask participants to prepare a place for installing the stove. On the next visit, made within 7 days of enrollment, the intervention facilitators will demonstrate the LPG stove and cylinder installation and inform enrolled women of the process for subsequent gas cylinder replenishment. In this visit, the intervention staff will counsel the women and other household members regarding safety measures and share the behaviour change communication material. The visits will continue monthly.

#### Improve adherence and monitor compliance

The monthly visit by the intervention staff is to support exclusive use of LPG cooking, ensure safety, and troubleshoot any issues. The tasks consist of observing the cooking place with a checklist (including looking for signs of biomass cooking), delivery of the behaviour change material, and monitor safety. If evidence of biomass fuel cooking is detected, our staff will discuss the reason and encourage the woman and her household to cook exclusively with LPG.

#### Management and training

A team of four field supervisors will monitor the ~70 surveillance staff and ten intervention staff and report to a field research manager to supervise all the field activities. We will train all staff in their respective duties for the household visits and pregnancy identification. Intervention staff will receive intensive training in installation and use of LPG, and behaviour change messages related to exclusive use of gas for cooking. We will conduct the training in phases, with regular refresher courses, and monthly meetings to discuss field-related troubleshooting. Supervisors will also provide daily feedback to the staff. The project management group meets weekly, reviews the conduct of the trial in detail as needed, and audits field activities routinely. There is no procedure for additional auditing.

#### Control arm

Study participants do not receive a cookstove or LPG cylinders in the control arm and continue their usual cooking practices. Usual cooking practices in this setting and most rural settings in Bangladesh involve a traditional clay stove with an open fire burning biomass fuel, usually crop waste and sticks. However, this fuel type and location vary by season and household income. Evidence of the effectiveness of improved stoves in the field suggested that they were unlikely to sufficiently reduce household air pollution and we therefore did not consider them an appropriate comparator [32]. Control participants will receive a small gift at the birth visit (an infant blanket).

### Outcomes and assessment of the outcomes

#### Primary outcomes

The primary outcome is the perinatal mortality rate difference between the intervention and the control arm. Perinatal mortality is a composite indicator, including the sum of the number of stillbirths (fetal death in pregnancy of at least 28 weeks gestation or more) and the number of early neonatal deaths (the death of a live-born infant up to and including 7 completed days of age of liveborn infants regardless of gestational age or birth weight) [25, 33].

#### Secondary outcomes

Our secondary outcomes include a comparison between the intervention and control arm in terms of (i) perinatal morbidity: preterm birth (birth before 37 completed weeks gestation or in the absence of gestation birth weight <2500g) and low birth weight (<2500g); (ii) cost-effectiveness of LPG in reducing perinatal mortality; (iii) personal-level exposure to household air pollution; (iv) satisfaction and acceptability of the LPG stove and stove use (per cent of cooking time with LPG stove); and (vi) cost-effectiveness and cost-utility of gas cooking compared to the usual cooking practice. We will also measure changes between groups at each time point in blood pressure, anaemia, and gestational diabetes. We anticipate that women in the LPG group will be less hypertensive and less anaemic; however, we do not expect differences in gestational diabetes.

**Fig. 3:**
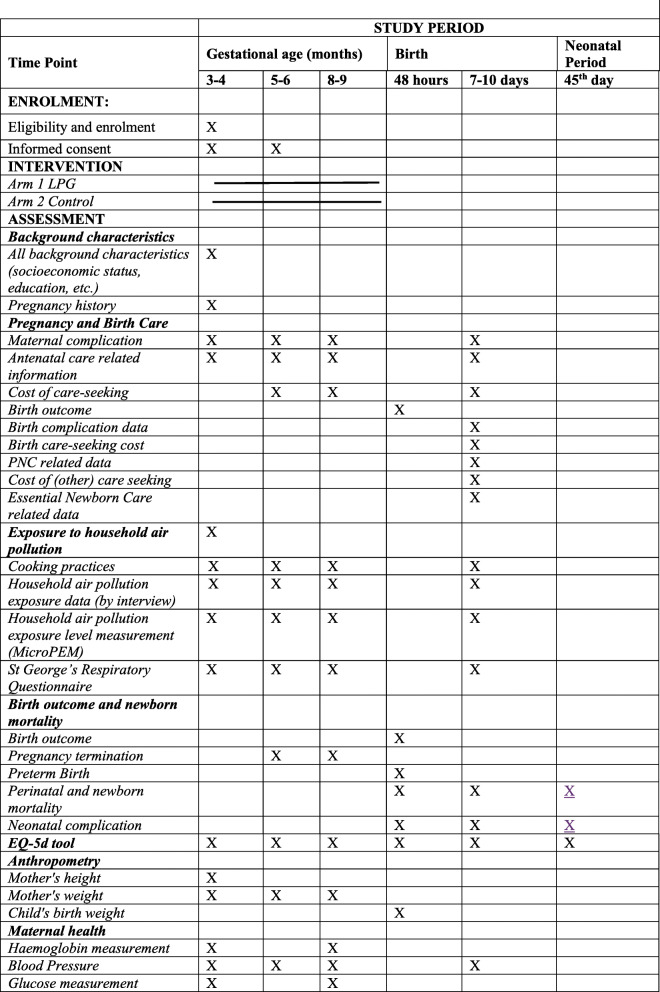
Schedule of data collection and trial activities for Poriborton

Types of birth outcomes (live or stillbirths) will be measured at the first post-partum follow-up visit within 48 h of birth (Fig. 3). If the enrolled woman is absent or the field team is not informed of the birth outcome, or the birth occurred beyond 48 h, the birth outcome will be collected at the 7–10 days or on 45th-day post-partum visits. Information on the occurrence of early neonatal deaths will be collected within 48 h, 7–10 days, and 45th day after birth depending on the timing and notification of the death. Data collection time points for secondary outcomes have been specified in Fig. 3.

### Exposure assessment of the particulate matter

Personal household air pollution exposure will be measured in a subset of 300 women equally divided between each arm. Data collection will occur at 3–4 months gestation, 5–6 months, and 8–9 months. We will use the MicroPEM (RTI, Research Triangle Park, NC) worn in the pocket of a sash located on the upper left of the women’s chest to measure personal exposure. The MicroPEM is a low burden and wearable (240 g with batteries) particulate matter (PM) exposure monitor. PM_2.5_ concentrations will be measured with a nephelometer and with a filter. Additional filter analysis will quantify the black carbon and tobacco smoke concentration [34]. We calculated the estimated participant compliance with wearing the MicroPEM from the accelerometer data [35]. Purple Air monitors deployed outdoors will measure ambient PM_2.5_ concentrations to separate HAP exposures from other exposure sources such as brick kilns or rice mills.

### Process evaluation for adequacy of the intervention

Data on cooking practices will be collected at baseline using a structured questionnaire and visual inspection (with a checklist) of the cooking environment, which we have used before [26, 36]. Adherence to LPG cooking is collected monthly in the intervention arm. This data will identify any contextual factors that influence (exclusive) use of LPG cooking [34]. Qualitative data is collected through interviews and focus groups of a purposively selected sample of intervention recipients (women and their household members) and all cadres of project staff.

### Sample size and power calculation

We calculated the sample size for the primary outcome, a reduction in the perinatal mortality rate in the intervention compared to the usual practice arm. A systematic review of polluting fuels versus clean fuels reported an elevated risk of perinatal mortality of 56% (aRR: 1.44, 95% *CI*: 1.30, 1.61) [37]. Therefore, we have calculated our sample size conservatively informed by this estimate, and the considered data from published studies at the time of developing the protocol [5, 8, 11, 12]. We assumed a 35% decrease in perinatal mortality (effect size) in the LPG arm. Our previous field work estimated perinatal mortality at 50 per 1000 pregnancies in control clusters [38]. We considered 80% power and 5% two-sided alpha and an average cluster size of 20 births. The published intra-class correlations for stillbirth and neonatal mortality in rural Bangladesh are very low, 0.00055 and 0.00000, respectively [39]; thus, we used a conservative intra-class correlation midway between the estimates of 0.000275 [39]. We used the standard formula [40] to estimate the number of births required for the study to be 4120 (2060 per arm) from a total of 206 clusters. We inflated the sample size to attain these births to 4944 pregnant women (2472 per arm) from a total of 206 clusters (24 women per cluster) considering 10% pregnancy loss (abortion/miscarriage < 28 weeks gestation) and another loss of ~5% of women who move and birth outside the study area.

### Sample size for the household air pollution exposure

We used the data from our feasibility study and a previous study conducted in Sri Lanka to estimate the sample size for the personal exposure measurements [41]. The total number needed is 300 women (150 per arm) to complete the personal exposure measurements at three gestational time points. This will demonstrate a reduction in household air pollution exposure resulting from the LPG intervention with an effect size of 0.5 and 95% power and 5% error.

### Data collected

We will collect demographic, socioeconomic, and household characteristics; birth history; birth outcomes; birthweight; maternal anthropometry; cost of care-seeking; cooking practices; and sources of household air pollution surveys from all participating women. We will also assess the haemoglobin and non-fasting glucose. These are collected with standard field practices using a drop of blood from a needle prick and measured with an hemocue or glucometer. Measurements will be taken at baseline and in the final trimester ~8–9 months gestation. We measure diastolic and systolic blood pressure using the CRADLE VSA at baseline, at 4–5 months, and at 8–9 months. Personal exposure to household air pollution will be collected as previously described. Figure 3 outlines the data collection schedule and data collection forms are available on request.

### Data collection procedures

A separate team of ~12 evaluation staff with no involvement in trial implementation will be responsible for collecting the outcome assessment data. All evaluation staff are female and recruited locally and supervised by four field supervisors. All evaluation staff will receive training for 2 weeks with regular refresher training. Training will include all aspects of data collection, including consent taking, different data collection methods, anthropometric measurements, blood pressure measurement, haemoglobin and glucose, household air pollution exposure measurement, electronic data capture, and relevant cultural considerations. We will provide supportive supervision for all staff on site.

### Electronic data collection tool

Data is captured electronically in the field using an android platform tablet with a specifically designed application (app). The app will record all surveillance and trial information. It will schedule visits for each household and prepare monthly visits and activity schedules for each data collector. The app reports all data back to the central database system in real-time, but the intervention and evaluation teams only have access to the data relevant to their position. The field research manager, overseen by staff in Dhaka, monitors all field staff visit schedules and data quality.

### Access to data

Data will be accessible by all investigators to analyse and publish, and we will only use the information from the respondents for research purposes. We will share data after removing participants’ personally identifiable information. All participant personal identifiers entered by the data collector will automatically be suppressed from the data collection form once the data is uploaded to the password-protected server.

### Data management

All electronic data is stored on a server backed-up at icddr,b and only limited staff can access it with passwords. Data will be stored for 15 years as per funder rules by the sponsor.

### Statistical analysis

We will conduct an intention-to-treat analysis and assess outcomes at the individual level. To evaluate the balance of key background and potential confounding variables, we will summarise the baseline characteristics by treatment group. To assess the impact of the intervention, we will compare perinatal mortality, preterm birth, and low birth weight between the intervention versus the control arm. Our analyses will estimate relative risks and 95% confidence intervals of point estimates using generalised estimating equations, logistic binomial regression models, a log link function, and exchangeable correlation [42]. The intervention will be a fixed effect, and the cluster as a random effect to account for clustering. We will do separate models for our specified outcomes. To ensure we only detect potentially important interactions with the mortality outcomes, we will increase power (*P* <0.10 (vs <0.05)) in the generalised estimating equation logistic regression models [43]. There are no interim analyses planned, and there are no stopping rules.

For the exposure data, we will calculate exposure-response functions for different exposure metrics, first using graphical summaries (e.g. loess plots) to examine the shape of the relationship of the exposure metrics to the outcome. For this initial exploratory stage, we will also use standard multivariate approaches (e.g. principal components, spectral decompositions) to reduce the dimensionality of high-dimensional metrics. Real-time measurements of PM_2.5_ will allow us to explore which summary metrics (e.g. integrated total mass exposure, peak exposure levels, and time above risk threshold) are most closely associated with the outcome. Based on the exploratory analyses, we will then use appropriate generalised linear models to generate estimates of the effect of the exposure metrics (or combinations of) on the outcomes. We will calculate the effect sizes and 95% confidence intervals for all analyses and present the exact *P*-value.

### Trial registration

We registered the study with the Australian New Zealand Clinical Trials Registry ACTRN12618001214224.

### Dissemination plan

We will share the trial findings with the stakeholders from Bangladesh and global audiences through a dissemination ceremony (if possible in person). We will also present the findings at conferences and publish in conference papers and international peer-reviewed journals. We will distribute the results using appropriate methods, such as community meetings. If the results are favourable to the intervention, we will work with relevant stakeholders to ensure access to the intervention for all communities, these discussions have already commenced. The datasets analysed during the current study and statistical code are available from the corresponding author on reasonable request, as is the full protocol.

This protocol has been written according to the recommendations of the SPIRIT 2013 statement [44] (Additional file 1).

### Adjustment to the procedures due to COVID-19 and status update

In the last week of March 2020, the Government of Bangladesh instigated a movement restriction intervention to curb SARS-COV-2 infections, effective from the 26th of March 2020. At that time, we had already finished our fifth round of household menstrual surveillance. We, therefore, stopped recruitment and decided not to enrol any new participants from the 26th of March. However, we continued household menstrual surveillance over the phone to allow the field team to quickly enrol eligible women once the restrictions were lifted. We also continued follow-up of the enrolled women over the phone. We made the necessary changes in the questionnaire; for instance, we added an additional question whether the visit was conducted physically or over the phone, and an extra option “Cannot be done as the interview was conducted over the phone” to the questions for maternal and newborn anthropometry. Our staff gave support for consistent and continued LPG use over the phone, and we sought clearance and were approved to continue LPG cylinder replacement during the lockdown.

We developed an Infection Prevention and Control protocol, based on WHO advice. We developed different infection protocols for each cadre of workers (i.e. office staff, surveillance workers, evaluation workers, intervention workers). Additionally, we are keeping a record of COVID-19 cases at the study site. Any staff who conduct home visits are also expected to adhere to a comprehensive Personal Protective Equipment protocol, and icddr,b has approved both protocols. All project staff received training on both protocols. From the 9th of August 2020, we resumed enrollment of participants, physical visits for data collection, and intervention facilitation. Field supervisors monitor the activities closely so that all protocols are strictly maintained. The field team reports to investigators on COVID-19 updates, the project staff’s infection control procedures, and participants (if any) every week. All staff were fully vaccinated in early 2021.

At the time of submission of the manuscript, all participants had been recruited and follow-up of the pregnant women was continuing and all births were expected to end in March 2022.

### Ethics approval and consent to participate

The Research Review Committee and the Ethical Review Committee of icddr,b (PR-17103), and the Human Research Ethics Committee of the University of Sydney, Australia (PR-2018/717), Australia, approved the study. We have gate-keeper consent from community leaders and relevant government officials. Evaluation staff will obtain informed consent to participate from all women and/or their guardians and will have the right to withdraw at any stage without penalty or loss. On the consent form, participants will be asked if they agree to the use of their data should they choose to withdraw from the trial. Participants will also be asked for permission for the research team to share relevant data with people from other institutions taking part in the research or from regulatory authorities, where relevant. This trial does involve collecting haemoglobin and non-fasting glucose. There will be no special criteria for discontinuing or modifying allocated interventions.

The investigators will ensure the privacy, anonymity, and confidentiality of the information provided by respondents and will store all trial information in an encrypted database with all identifiers removed. Poriborton is not considered a health intervention as such and the local ethical review committee at icddr,b considered this a low-risk project and did not recommend a data monitoring committee necessary. We will report any serious adverse event or unanticipated problems of potential risk for study participants or others using a standard case reporting form to icddr,b’s ethics review committee within 24 h of occurrence. Any changes to the protocol will be communicated to the committees above and the trial registry.

## Discussion

This trial will provide high-level evidence of the effect of reduced exposure to household air pollution from cooking with LPG on pregnancy outcomes, specifically perinatal mortality and morbidity. Until now, evidence of the effect on perinatal mortality has been mostly observational, retrospective, and/or using secondary data, and for perinatal studies, neither the exposure nor perinatal mortality has been measured accurately and, or prospectively, leaving uncertainty around the estimates [9]. Accurately measuring perinatal morality even in high-burden countries requires a prospective design, with an accurate case definition that considers the context of the setting and is sufficiently powered to detect a difference. Small, retrospective studies are generally insufficiently powered, and more importantly, are subject to recall bias.

The Government of Bangladesh released a strategic plan for LPG distribution, and it declared a target of 70% for all households to use LPG by 2030. They previously set access to electricity targets for 2021, but these did not cover cooking fuels [45, 46] (https://data.worldbank.org/indicator/EG.ELC.ACCS.ZS?locations=BD). Clean or cleaner fuel targets are important for health and the environment and Goal Seven of the Sustainable Development Goals (https://www.un.org/sustainabledevelopment/energy/) includes such targets. This trial will provide evidence of the impact of household air pollution on perinatal outcomes.

## Trial status

Protocol version 1 (19 July 2019). Recruitment commenced in Sept 2019 and ended in June 2021, and follow-up is expected to be complete in March 2022. There have been several hurdles in running the trial due to the pandemic, including confirmed SARS-CoV-2 infections in our implementing team and movement restriction orders placed on staff in three countries (Bangladesh, Australia, and the USA). These and the changes to our roles and responsibilities due to these movement restrictions (home-schooling children, online teaching, increased workload, inability to travel to the study site, etc.) have adversely impacted our available time and progress, and hence, we submit this protocol after the recruitment was completed.

## Supplementary Information

**Additional file 1.** SPIRIT checklist

## Data Availability

Any data required to support the protocol can be supplied on request.
