# Supplementary material for: Protocol for a cluster randomised controlled trial of LPG cookstoves compared to usual cooking practices to reduce perinatal mortality and morbidity in rural Bangladesh called Poriborton: the CHANge trial
Source: Trials. 2022 Apr 18;23:325. doi: 10.1186/s13063-022-06146-7 (PMC9014282; doi:10.1186/s13063-022-06146-7)
Supplement: Supplementary file 1 — Additional file 1. SPIRIT checklist [file 13063_2022_6146_MOESM1_ESM.docx]

**Poriborton Protocol – Trials TRLS-D-21-01194 - [EMID:34e089605ea28593]**

Please include:

- a point-by-point response within the 'Response to Reviewers' box in the submission system
- revised manuscript with tracked changes

**Due date 5^th^ March 2022**

| Item 4:  The authors simply state, line 490: "Neither the sponsor or the funders have any role or decisions in any activities." Could they please state the activities, assuming correct that: "The sponsor and funding body played no role in the design of the study and collection, analysis, and interpretation of data and in writing the manuscript." | Text amended on page 24, line 509:  “Neither the sponsor or the funders played have any role or made any decisions regarding the intervention, in any activities nor did they play a role in the design of the study, or the collection, analysis, and interpretation of data and in writing the manuscript.” |
| --- | --- |
| Item 5d:  This cannot be N/A. Perhaps you could give information on the composition, roles and responsibilities of the coordinating centre and trial steering committee and all groups providing day to day support for the trial. We also need information on who is responsible for all aspects of local organisation including identifying potential recruits and taking consent. Who is supervising the trial and how often they will meet, plus information on the Trial Steering Committee (TSC), and how often they will meet over the course of the trial to oversee conduct and progress. Please expand the information in the SPIRIT checklist and put it into the protocol, and a page number in the SPIRIT checklist. Plus, information and how often the Stakeholder and Public Involvement Group (SPIG) if there is one, and their specific role. | Text has been added on page 24, line 516:  “Principal Investigators and field management staff meet weekly via zoom to manage trial coordination, oversee participant safety, study design, database integrity, and study conduct. CIA Raynes-Greenow chairs the meeting, also attending weekly are CI Alam, CI Billah and the project Managers in Bangladesh; Assistant Scientists Islam, Rokonuzzaman, and Ferdous. There are no other committees responsible for this trial.” |
| Item 8:  Please can the authors include a description of trial design including the framework (e.g., superiority, equivalence, non-inferiority, exploratory). | Trial framework has been added on page 6, line 111:  “The design is a parallel community-based cluster randomised controlled superiority trial (cRCT) of two arms” |
| Item 11b:  Perhaps the authors can state: "There will be no special criteria for discontinuing or modifying allocated interventions". | Text has been added on page 22, Line 453:  “There will be no special criteria for discontinuing or modifying allocated interventions” |
| Item 12:  Could the authors make it clearer the time points for measurement of the composite primary outcome - perinatal mortality as per SPIRIT guidance. Think 28 days or more fetal death and also 6 days of age for live born infants. Is this correct? Please also make it clear when all secondary outcomes are measured. | We have added “liveborn infants” for early neonatal deaths on page 14, line 277  Text now reads:  “The primary outcome is the perinatal mortality rate difference between the intervention and the control arm. Perinatal mortality is a composite indicator, including the sum of the number of stillbirths (fetal death in pregnancy of at least 28 weeks gestation or more) and the number of early neonatal deaths (the death of a live-born infant up to and including six days of age of liveborn infants regardless of gestational age or birth weight)”  Secondary outcomes are measured at different timepoints specified in figure 3. We have added following text on page 14, line 288  Text reads:  “We capture the pregnancy outcome data in both study arms within 48 hours of birth, perinatal mortality up to 15 days after the birth, and neonatal deaths up to 45 days after the birth. Data collection time points for secondary outcomes have been specified in figure 3” |
| Item 15:  Recruitment can never be Not Applicable. I am delighted the authors understood that there would not be an issue with recruitment but they still have to give information for this SPIRIT item, as do all authors submitting protocols to Trials. Please insert into the protocol information on recruitment strategy to ensure adequate participant enrolment to reach sample size. You need to state in the protocol how you will recruit and for instance the recruitment period. Where and extra measures taken to recruit sufficient number.  This information should be placed in this SPIRIT item 18b - "control participants receive a small gift" | We had described participant recruitment process in page 11, lines 215-228.  We have added following text on page 12, line 230:  “Our door-to-door surveillance of ~92,000 households over a period of 12 months is expected to identify and enrol required number pregnant women within 120 days of gestational age.”  This text “control participants receive a small gift (Page 13, line 262)” has been moved to SPIRIT item 18b |
| Item 17a:  The authors could include information in the protocol stating that blinding was not feasible for women in the clusters and for the intervention of LPG stove and cylinder distribution. However, the outcome assessment is objective and collected in electronic data. I am not clear if data analysts are blinded. | Text added on page 8, line 140  “To minimise bias, because participant blinding is not possible, we will have separate intervention and evaluation staff. Trial evaluators will be blinded to the hypothesis, and we will conduct our analysis blinded to the treatment arms.”  Yes, the data analysts are blinded – see Item 17b |
| Item 17b:  N/A is not acceptable please write in the SPIRIT checklist "the design is open label with only data analysts being blinded (if correct) so unblinding will not occur | Amended in Item 17b, page 39:  “The design is open label with only data analysts being blinded so unblinding will not occur.” |
| Item 18b:  Please complete and detail plans to promote participant retention and compete follow up, including list of any outcome data to be collected for participants who discontinue or deviate from intervention protocols. Insert information into the protocol with page number in the checklist.  The following statement should be placed in this SPIRIT item - "control participants receive a small gift". The response is ITT is insufficient. | Intention to treat analysis will be conducted.  Text added page 18, Line 374  The text has been moved as suggested. |
| Item 21a:  I could not find this information on page 21 under Ethics Approval and consent to participate. Under this item, please state composition of data monitoring committee; summary of its role and reporting structure; statement of whether it is independent from the sponsor and competing interests.  Alternatively, an explanation of why a DMC if not needed. This requires information inserted into the SPIRIT checklist on Data Monitoring committee referring to page X or perhaps stating that additional information is in the Appendix. | Text amended to read (from page 22, line 457):  “Poriborton is not considered a health intervention as such and the local ethical review committee at icddr,b did not deem a DMC necessary. We will report any Serious Adverse Event or unanticipated problems of potential risk for study participants or others using a standard case reporting form to icddr,b’s ethics review committee within 24 hours of occurrence. Relevant committees will monitor any events and as this is a low-risk intervention, and based on local advice, we will not establish a formal data monitoring committee. “ |
| Item 23:  Frequency and plans for auditing trial conduct - needs information, this cannot be N/A.  For instance, how often Project Management Group meet to review trial conduct. The Trial Steering Group and the independent Data Monitoring and Ethics Committee meet to review conduct throughout the trial period.  It may be that a Data Monitoring Committee was not considered as this was a low-risk intervention, in which case please state this in the protocol and put a page number into the SPIRIT checklist. | Text added to line page 13, line 258:  “Project management group meets weekly, and reviews conduct of trial in detail as needed. There is no procedure for additional auditing.”  Text re data monitoring committee was added on page 22 Line 457. |
| Item 26a:  This SPIRIT item request information on who was actually recruiting the patient and gaining informed consent. When and how is this done? I believe the information is earlier in the manuscript on page 18, line 342 when the authors mention training of "evaluation staff with no involvement in trial implementation" and training will include "consent taking".  On page 21 it says who is giving consent but not who is actually recruiting and gaining informed consent. On line 412 there is a mention of field team enrolling eligible women. Please edit to make this clear. | Text on page 21, line 447 amended to:  “Evaluation staff will obtain informed consent to participate from all women and or their guardians provide informed consent to participate and will have the right to withdraw at any stage without penalty or loss.” |
| Item 26b:  Cannot just say "Same consent as above." This particularly concerns biological samples - haemoglobin and non-fasting glucose. Can suggest something like this "On the consent form, participants will be asked if they agree to use of their data should they choose to withdraw from the trial. Participants will also be asked for permission for the research team to share relevant data with people from the Universities taking part in the research or from regulatory authorities, where relevant. This trial does involves collecting haemoglobin and non-fasting glucose." | Text has been added as suggested to page 21, line 449:  “On the consent form, participants will be asked if they agree to use of their data should they choose to withdraw from the trial. Participants will also be asked for permission for the research team to share relevant data with people from the Universities taking part in the research or from regulatory authorities, where relevant. This trial does involve collecting haemoglobin and non-fasting glucose.” |
| Item 29:  Consider stating "Any data required to support the protocol can be supplied on request." | This text has been added to page 24, line 499. |
| Item 31b:  page 24 lines 496-500 you state the author contributions please insert page number into checklist and please also state "All named authors adhere to the authorship guidelines of Trials. All authors have agreed to publication." Or "All authors have contributed to writing the manuscript as detailed p24, no professional writers have been involved." | On page 25, line 527 additional text added as suggested.  “All named authors adhere to the authorship guidelines of Trials. All authors have agreed to publication” |
| Item 31c:  Access to data must be completed - it is really important so complete information.  Item 31 c: I wonder whether the authors would consider changing their statement data - "Not relevant".  Please consider stating that: "The datasets analysed during the current study and statistical code are available from the corresponding author on reasonable request, as is the full protocol." | The following text has been added to page 20 line 409:  “The datasets analysed during the current study and statistical code are available from the corresponding author on reasonable request, as is the full protocol.” |
| Item 33:  For this SPIRIT item, please add information on plans for collection, laboratory evaluation, and storage of biological specimens for genetic or molecular analysis in the current trials and for future use in ancillary studies." | N/A as we will not collect biological specimens for this study. |

References:

There were 18 articles that could not be checked. One article was not validated - https://protect-au.mimecast.com/s/9Mk3CP7LAXfKgjJZKiBfLKo?domain=trialsjournal.biomedcentral.com

Please also take a moment to check our website at https://protect-au.mimecast.com/s/mrpeCQnMBZfkV2J9ktQe5LS?domain=editorialmanager.com for any additional comments that were saved as attachments. Please note that as Trials has a policy of open peer review, you will be able to see the names of the reviewers. If your manuscript is published in the journal, the reviewer reports and your responses to the reviewers will be published alongside the article.
